# *DNAJC30* Gene Variants Are a Frequent Cause of a Rare Disease: Leber Hereditary Optic Neuropathy in Polish Patients

**DOI:** 10.3390/ijms242417496

**Published:** 2023-12-15

**Authors:** Anna Skorczyk-Werner, Katarzyna Tońska, Aleksandra Maciejczuk, Katarzyna Nowomiejska, Magdalena Korwin, Monika Ołdak, Anna Wawrocka, Maciej R. Krawczyński

**Affiliations:** 1 Department of Medical Genetics, Poznan University of Medical Sciences, 60-806 Poznan, Poland; ania.wawrocka@gmail.com (A.W.); mrkrawcz@ump.edu.pl (M.R.K.); 2 Institute of Genetics and Biotechnology, Faculty of Biology, University of Warsaw, 02-106 Warsaw, Poland; k.tonska@uw.edu.pl (K.T.); a.maciejczuk@student.uw.edu.pl (A.M.); 3 Department of General and Pediatric Ophthalmology, Medical University of Lublin, 20-079 Lublin, Poland; katarzyna.nowomiejska@umlub.pl; 4 Department of Ophthalmology, Medical University of Warsaw, 02-005 Warsaw, Poland; magdalenakorwin@gmail.com; 5 Department of Genetics, Institute of Physiology and Pathology of Hearing, 02-042 Warsaw, Poland; monika.oldak@wum.edu.pl; 6 Department of Histology and Embryology, Center of Biostructure Research, Medical University of Warsaw, 02-004 Warsaw, Poland; 7 Center for Medical Genetics GENESIS, 60-529 Poznan, Poland

**Keywords:** Leber hereditary optic neuropathy (LHON), autosomal recessive LHON (LHONAR; arLHON), *DNAJC30*, mtDNA (mitochondrial DNA)

## Abstract

Leber hereditary optic neuropathy (LHON) is a rare disorder causing a sudden painless loss of visual acuity in one or both eyes, affecting young males in their second to third decade of life. The molecular background of the LHON is up to 90%, genetically defined by a point mutation in mitochondrial DNA. Recently, an autosomal recessive form of LHON (LHONAR1, arLHON) has been discovered, caused by biallelic variants in the *DNAJC30* gene. This study provides the results of the *DNAJC30* gene analysis in a large group of 46 Polish patients diagnosed with LHON, together with the clinical characterization of the disease. The c.152A>G (p.Tyr51Cys) substitution in the *DNAJC30* gene was detected in all the patients as homozygote or compound heterozygote. Moreover, we identified one novel variant, c.293A>G, p.(Tyr98Cys), as well as two ultra-rare *DNAJC30* variants: c.293A>C, p.(Tyr98Ser), identified to date only in one individual affected with LHONAR1, and c.130_131delTC (p.Ser44ValfsTer8), previously described only in two patients with Leigh syndrome. The patients presented here represent the largest group of subjects with *DNAJC30* gene mutations described to date. Based on our data, the autosomal recessive form of LHON caused by *DNAJC30* gene mutations is more frequent than the mitochondrial form in Polish patients. The results of our study suggest that Sanger sequencing of the single-exon *DNAJC30* gene should be a method of choice applied to identify a molecular background of clinically confirmed LHON in Polish patients. This approach will help to reduce the costs of molecular testing.

## 1. Introduction

Leber hereditary optic neuropathy (LHON, MIM: #535000; ORPHA: #104) is a rare disorder manifested by a sudden painless loss of visual acuity in one or both eyes. The disease has a strong male preponderance (80–90%) [1]. LHON mainly affects young males in their second to third decade of life, usually between ages 15 and 35, but symptoms can also be seen in childhood or at an older age [1,2]. The prevalence of the disease is about 1:30,000 in Europe. LHON displays a sex-dependent incomplete penetrance higher in men (about 50%) than in women (10%) [3,4].

LHON is a neurodegenerative disease of the optic nerve, causing the initial dysfunction of retinal ganglion cells, followed by axon degeneration. The first symptoms of the disease include a sudden acute or subacute painless, unilateral loss of central vision with involvement of the other eye after a few weeks to months later or affecting both eyes simultaneously. Visual acuity loss generally stabilizes within 4–6 months, and in most patients, the visual acuity is worse than 20/200. Visual field defects are typically central or cecocentral. Most patients observe central scotoma, usually at the early stage of the disease, but later, visual field defects become larger. Among the symptoms of LHON, there is also the early impairment of color perception [1,5].

During the first 4–5 weeks of the disease, which is called the acute phase, the eye fundus and OCT examinations usually reveal an elevation of the optic nerve disc and perivascular telangiectatic microangiopathy, as well as the swelling of the retinal nerve fiber layer, which, unfortunately, is often mistakenly considered a sign of an inflammatory process. In approximately 20% of patients, the eye fundus may look normal during the acute phase of LHON, which can delay the accurate diagnosis [6]. Eventually, the signs of the chronic phase become apparent after a few months. Funduscopy and OCT reveal optic disc pallor, thinning, and the atrophy of the retinal nerve fiber layer and ganglion cell complex at this phase of LHON. The disease progresses rapidly, with only a negligible probability of visual recovery depending on the type of mitochondrial mutation [2,4]. However, in more than 45% of patients, visual acuity improved after being treated with idebenone, a short-chain synthetic ubiquinone analog [7,8].

Leber hereditary optic neuropathy is known to be the most frequent mitochondrial disease. The molecular background of the LHON is up to 90%, genetically defined by a point mutation in mitochondrial DNA. The vast majority of mitochondrial LHON (mtLHON) cases are caused by one of three primary mitochondrial mutations within the genes encoding mitochondrial complex I subunits (NADH–ubiquinone oxidoreductase): m.11778G>A (localized in the MT-ND4 gene), m.3460G>A (within the MT-ND1 gene), and m.14484T>C (within the MT-ND6 gene) [2,4].

Recently, an autosomal recessive form of Leber hereditary optic neuropathy (LHONAR1, arLHON, MIM: #619382) has been discovered. The autosomal recessive form of LHON is mainly caused by biallelic variants in the *DNAJC30* (MIM: 618202) gene [8,9]. Typical LHON phenotypes have also been reported to be the effects of variants in other genes, namely *NDUFS2*, *MCAT,* and *NDUFA12*, detected only in a few families [10,11,12,13]. Pathogenic variants in the *DNAJC30* gene were reported to affect the mitochondrial complex I subunit and interact with the mitochondrial adenosine triphosphate (ATP) synthesis [9].

The *DNAJC30* encoding the DNAJ/HSP40 homolog, subfamily C, member 30 protein, controls ATP synthase activity and plays a leading role in the repair mechanism of oxidative phosphorylation complex I. DNAJC30 is a chaperone protein expressed mainly in neurons. It is required to efficiently exchange complex I subunits exposed to reactive oxygen species and integral to a mitochondrial complex I repair mechanism [9]. The affected DNAJC30 protein is incapable of regenerating the damaged complex I subunits. It results in low ATP synthesis and high levels of reactive oxygen species, which causes the degeneration of retinal ganglion cells. Similarly to mtLHON, the autosomal recessive form of LHON also demonstrates sex-dependent incomplete penetrance with more affected men than women [9].

About 100 LHON patients with *DNAJC30* mutations have been reported [8,9,10]. Approximately 85% of patients originate from Eastern Europe (Russia, Ukraine, Poland, and Romania). Most patients were affected with the c.152A>G variant, causing the amino acid substitution of tyrosine at position 51 within the conservative domain of DNAJC30 protein to cysteine. The p.Tyr51Cys substitution is a founder mutation in Eastern Europe [9].

From the year 2008 until April 2021, the routinely performed genetic testing in Polish patients with suspected Leber hereditary optic neuropathy was basically restricted to Sanger sequencing of three mtDNA regions encompassing three primary LHON-causing mutations: m.11778G>A, m.3460G>A, and m.14484T>C. Some patients with no mtDNA mutations detected based on this diagnostic strategy had whole mitochondrial genome sequencing performed. Moreover, the DNA sequencing of the genes associated with optic atrophies was performed in some individuals.

In April 2021, following the report on the *DNAJC30* gene variants as a cause of the autosomal recessive form of LHON [9], we implemented molecular testing for *DNAJC30* variants in those patients in whom no changes in the mtDNA were detected. Although it is known that mtDNA mutations cause most LHON cases, many Polish patients with the typical course of the disease have been waiting for a molecular diagnosis for many years until we implemented the *DNAJC30* gene sequencing.

This study aimed to report a clinical characterization and molecular basis of the autosomal recessive form of Leber hereditary optic neuropathy in 46 Polish patients, the largest cohort of patients with the LHONAR1 reported to date. Based on our data, the autosomal recessive LHON caused by *DNAJC30* gene mutations is more frequent than the mitochondrial form in Polish patients. Moreover, by reporting a novel variant, this study broadens the mutation spectrum of probable pathogenic *DNAJC30* variants. In this paper, we also show the diagnostic odyssey undergone by some of our patients, which is no longer needed since a simple, inexpensive molecular test for variants’ screening in one small gene enables establishing a molecular diagnosis in most Polish LHON patients.

## 2. Results

From April 2021, all the patients with no mtDNA variants detected and those referred to our clinic for the first time were offered molecular testing for *DNAJC30* gene variants. Here, we present the results of the *DNAJC30* gene analysis in a large group of 46 Polish patients clinically diagnosed with Leber hereditary optic neuropathy.

### 2.1. Clinical Diagnosis of Leber Hereditary Optic Neuropathy

Most patients’ first symptom of LHON was the deterioration or loss of visual acuity. It mostly involved one eye first, followed by the other eye over three days to two years (usually, the fellow eye was affected after two weeks to three months). The average age of LHON onset among our patients was 20.5 years (19.5 years, excluding patient no. 40_45, whose age of onset was 68). The average age of onset among affected women was the same as that in a group of men (when excluding patient no. 40_45) and was equal to 19 years. Fundus examination showed hyperemic optic nerves during the acute phase of LHON in most patients. Moreover, peripapillary microangiopathy was observed at the beginning of the disease in many patients. Most patients reported centrocecal scotoma or other visual field defects, and some patients noticed impaired color perception. Table 1 presents the clinical symptoms observed in the 46 patients.

### 2.2. Molecular Genetic Analysis

The sequencing analysis of the three mtDNA regions encompassing three primary LHON-causing mutations performed in one proband from each family (40 subjects), excluding patient no. 23_26 from family 23, did not reveal any causative mitochondrial DNA variants. Twenty-two patients (patients’ ID numbers are listed in Materials and Methods) with no variants found in the three analyzed mtDNA fragments were subjected to whole mitochondrial genome sequencing, which did not lead to the detection of any potentially pathogenic variants. Three benign variants in mitochondrial DNA were identified in three patients: mt.11253T>C (in patient no. 3_3), mt.14199T>C (in the patient no. 8_9), and mt.11654A>G (in patient no. 16_18).

Before the *DNAJC30* gene screening was implemented in diagnostics, some patients from our study group underwent additional molecular tests. Multiplex ligation-dependent probe amplification (MLPA), performed in patients no. 15_17, 18_20, and 21_24, failed to identify the molecular background of the disease. Single-nucleotide polymorphism (SNP) microarray based on APEX technology for detecting variants in the *OPA1* gene carried out in patients no. 15_17 and 18_20 also did not reveal any causative variants. Moreover, a next-generation sequencing (NGS) panel encompassing genes associated with optic atrophy (excluding the *DNAJC30* gene at that time) and an NGS panel for genetically determined retinal diseases encompassing 274 genes performed in patient no. 15_17 did not detect any potentially pathogenic variants.

Patients with the still unknown molecular basis of LHON were subjected to Sanger sequencing of the *DNAJC30* gene (all patients excluding patients no. 20_22, 23_26, and 40_45) or the NGS panel encompassing the genes associated with optic nerve atrophy that included this gene (patients no. 20_22, 23_26, and 40_45). The recurrent variant c.152A>G (p.Tyr51Cys) was identified in all the examined patients, either in the homozygous state (in 41 patients) or in the form of compound heterozygote along with another variant on a second allele (in 5 patients).

**Figure 1:**
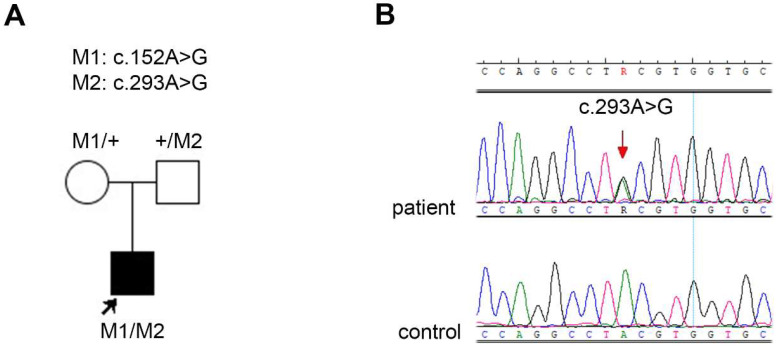
(**A**) Pedigree of the family 35 together with the segregation analysis results. The proband (patient no. 35_40) is marked with an arrow and a square filled with black. Unfilled symbols indicate unaffected, heterozygous parents. (**B**) Chromatogram showing the novel variant c.293A>G (indicated with the red arrow) in patient no. 35_40.

We discovered a novel variant, c.293A>G, p.(Tyr98Cys), in patient no. 35_40. The results of the segregation analysis in the family were consistent with the autosomal recessive mode of inheritance (see the pedigree and chromatogram in Figure 1).

**Figure 2:**
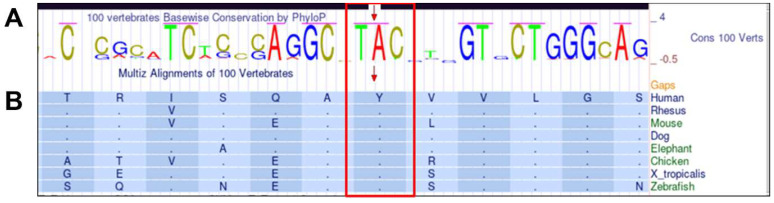
The conservation of the novel variant c.293A>G, p.(Tyr98Cys) based on the UCSC Genome Browser. The upper panel, (**A**), shows PhyloP results presenting the conservation of adenine and TAC codon in 100 vertebrates, and the lower panel, (**B**), shows Multiz alignments of amino acid tyrosine at position 98 conservation between selected vertebrate species.

The variant was not reported in the following databases Leiden Open Variation Database (LOVD, http://www.lovd.nl/3.0/home, accessed on 10 May 2023, Varsome (Varsome The Human Genomics Community, https://varsome.com/, accessed on 10 May 2023), ClinVar (https://www.ncbi.nlm.nih.gov/clinvar, accessed on 10 May 2023), and GnomAD Browser (Genome Aggregation Database, https://gnomad.broadinstitute.org/, accessed on 10 May 2023). According to the American College of Medical Genetics and Genomics (ACMG) classification guidelines, the variant is predicted to be pathogenic. The nucleotide adenine at position 293 and the codon TAC are conserved between species (Figure 2A). The p.(Tyr98Cys) variant is located within the conserved region of the protein, the J domain, which is crucial for the functional interactions of the DNAJC30 protein. Moreover, tyrosine at the amino acid position 98 is highly conserved between species (Figure 2B). The in silico predictions of the variant’s potential pathogenicity with the use of SIFT (score: <0.05, deleterious) and PolyPhen-2 (score: 1, probably damaging) indicated that the substitution is damaging.

Interestingly, we identified a different substitution at the same nucleotide position, an ultra-rare variant c.293A>C, p.(Tyr98Ser). We detected this variant in a compound heterozygous state in two brothers (patients no. 28_31 and 28_32). According to ACMG classification guidelines, the variant is predicted to be likely pathogenic. The in silico predictions of the p.(Tyr98Ser) potential pathogenicity using SIFT indicated that the substitution affects protein function (score: 0, deleterious). Our analysis with PolyPhen-2 showed that p.(Tyr98Ser) is probably damaging (score: 0.99). To the best of our knowledge, the substitution c.293A>C was only reported once in the ClinVar database in one patient affected with autosomal recessive Leber hereditary optic neuropathy (VCV001299584.1). The variant was identified in the GnomAD Browser as a heterozygote in 1 out of the 249,566 analyzed alleles in healthy individuals.

Moreover, in two patients, we identified an extremely rare deletion in the *DNAJC30* gene: c.130_131delTC (p.Ser44ValfsTer8). In both affected men, no. 1_1 and no. 22_25, the deletion was identified in a compound heterozygote state with c.152A>G (p.Tyr51Cys). This frameshift variant was classified as likely pathogenic. The substitution was previously identified in patients with Leigh syndrome: in one Polish family [14] and a Moroccan boy [15].

All *DNAJC30* gene variants identified in our patients are listed in Table 2.

## 3. Discussion

Here, we report the *DNAJC30* gene variants in 46 Polish patients with a clinical diagnosis of LHON. This is the first report on *DNAJC30* variants in such a significant cohort of patients, especially since they come from one country. To date, the autosomal recessive form of LHON has been diagnosed in about one hundred patients, most of whom were affected with *DNAJC30* gene variants [10].

Ophthalmological findings in our group of patients with autosomal recessive LHON were indistinguishable from those of the mitochondrial LHON form. The disease course was typical for LHON, usually involving acute painless central vision loss and chronic phase. No patients in our study group presented extraocular symptoms of LHON (LHON plus).

The average age of disease onset was 19 years when assessing both sexes together, as well as when assessing men and women separately. It is consistent with the already published data for men but differs from the reported data for women. Leaners and coworkers reported that the age of the autosomal recessive form of LHON onset in 10 affected women was higher than in our female patients and amounted to 32 years [10]. In our female patients, the disease began earlier, as the youngest patient was six years old at the age of LHONAR1 onset, and the oldest was 29. Interestingly, in this study, we also present a patient with very late disease onset, patient no. 40_45, who had first symptoms at the age of 68 years. The appearance of the first symptoms of Leber hereditary optic neuropathy in patients over 50 years of age is rare. However, cases of the mitochondrial LHON form with late disease onset have been reported [16,17,18]. It is worth noting that, to our knowledge, patient no. 40_45 is the first to be reported with an autosomal recessive form of LHON with such a late onset of the disease.

Diagnostic difficulties concerned not only this patient but also those patients in our study in whom, two or more years ago, no mitochondrial variants were identified. Over all these years, before the autosomal recessive form of LHON was reported, many patients with the typical course of LHON remained undiagnosed. These patients underwent a veritable diagnostic odyssey, waiting for a molecular diagnosis for even ten years. Some of them have had a large number of molecular tests performed, e.g., patient no. 15_17, who underwent MLPA and SNP microarray based on APEX technology for the *OPA1* gene as well as the NGS panel encompassing the genes associated with autosomal dominant optic atrophy and the NGS panel for genetically determined retinal diseases (without the *DNAJC30* gene included at that time). Moreover, the clinical diagnosis of LHON is complicated, especially if the patient is examined for the first time in the chronic phase of the disease when the clinical picture of the disorder resembles optic nerve atrophy caused by many other factors.

The other group of patients in the presented cohort are those affected with LHON who came to the genetic clinic within the last two years. Since the *DNAJC30*-related LHONAR was reported, the molecular diagnostic approach in patients with clinical features of LHON is relatively uniform in Polish patients. The most common procedure involves searching for the three most common variants in mtDNA or the whole mitochondrial genome sequencing and Sanger sequencing of the single-exon *DNAJC30* gene coding region. Nevertheless, three of the described patients had the NGS panel for genetically determined retinal diseases (encompassing 274 genes, including the *DNAJC30* gene), which was performed instead of the *DNAJC30* gene sequencing due to the suggestion of different optic nerve atrophy clinical diagnosis or due to the patient’s will. However, Sanger sequencing of the *DNAJC30* gene coding sequence is the low-cost, fast, and effective method in searching for the molecular background of LHON in Polish patients, suggesting that it should be implemented as the first-step test.

From 2008 to April 2023, we diagnosed the mitochondrial form of LHON in 102 patients examined in one of the genetic centers where molecular tests for LHON reported in this study were carried out. On the other hand, since we introduced the *DNAJC30* gene test two years ago, we diagnosed LHONAR1 in 32 patients (out of a described group of 46) in this genetic center. Comparing these data, we can conclude that the autosomal recessive form of LHON is much more frequent in Polish patients than the classic mitochondrial form. The relatively high prevalence of *DNAJC30* variants in patients with LHON supports the thesis that the *DNAJC30* gene analysis should be the test of choice in Polish patients with LHON.

The rapid molecular diagnosis of patients with LHON is crucial to undertake fast treatment, which is necessary to stop the process of optic nerve degeneration. Unfortunately, we have scarce data on the number of patients who applied Idebenon treatment and the effects of treatment in our group of patients. This results from the fact that many patients do not make follow-up appointments in the genetic clinic, and some do not even have a routine ophthalmic examination once their vision is stabilized.

The c.152A>G (p.Tyr51Cys) substitution is known to be the most common variant in the *DNAJC30* gene and a founder mutation. This substitution accounts for over 20% of molecularly diagnosed patients in Russia [9] and 90% of disease-associated variants in the German cohort. Interestingly, our research shows that the p.Tyr51Cys variant is widespread in Polish patients, as it was identified in all subjects with a frequency of nearly 95% of alleles. Moreover, we identified a novel variant c.293A>G, p.(Tyr98Cys) within the conserved region of the protein crucial for the DNAJC30 protein interactions. Functional analyses are required to explain the impact of the novel variant and two other variants detected in our patients, p.(Tyr98Ser) and p.(Ser44ValfsTer8), on the DNAJC30 protein function. No genotype–phenotype correlations were found in our cohort of patients.

## 4. Material and Methods

### 4.1. Patients

This report focuses on 46 patients from 41 Polish families with clinical symptoms of Leber hereditary optic neuropathy, in whom no mtDNA variants were identified. Still, the *DNAJC30* gene screening fully confirmed the clinical diagnosis. There were 41 affected men and 5 women in our study group. The age of disease onset in this group of patients with LHON was 6 to 68 years old.

The patients were numbered with Patient ID, where the first digit indicated the family number, and the next digit after the underscore character was the individual’s laboratory number. This study was conducted by the tenets of the Declaration of Helsinki and the Association for Research in Vision and Ophthalmology (ARVO) statement on human subjects. Written informed consent was obtained from all participants or their parents or legal guardians if patients were under 16 years old.

### 4.2. Molecular Analysis

#### 4.2.1. Mitochondrial DNA Analyses

The sequencing analysis of three mtDNA regions encompassing three primary LHON-causing mutations, namely m.11778G>A, m.3460G>A, and m.14484T>C, was performed in one proband from each family (40 subjects), excluding family 23 (patient no. 23_26). The primers used for amplification and sequencing, and the polymerase chain reaction (PCR) conditions are available upon request.

Until April 2021, the whole mitochondrial genome sequencing was performed in 22 patients, in whom no variants were detected in the previously analyzed mtDNA regions based on Sanger sequencing (patients no. 1_1; 2_2; 3_3; 14_16; 15_17; 16_18, 17_19, 18_20, 19_21, 20_22, 22_25, 24_27, 25_28, 26_29, 27_30, 28_31, 29_33, 30_34, 31_35, 32_37, 33_38, and 34_39). Mitochondrial DNA for next-generation sequencing was amplified in one fragment encompassing the whole molecule or, in case of lower DNA quality, in two overlapping fragments. Genomic libraries were created using the Nextera XT DNA Sample Preparation Kit and Nextera XT Index Kit (Illumina Inc., Foster City, CA, USA) and then sequenced using NGS technology on the MiSeq platform and the MiSeq Reagent Kit v3 (Illumina). The obtained data were subjected to bioinformatics analysis, aligned to the reference mtDNA sequence, rCRS, and subjected to variant identification using the CLC Genomics Workbench (CLC bio) software (v11). Variant pathogenicity was assessed using the MITOMAP database (https://www.mitomap.org/MITOMAP, accessed on 1 March 2021). Patients who came to the genetic clinic after April 2021 did not have whole mitochondrial genome sequencing performed. For details on the whole mitochondrial genome sequencing procedure, see Piotrowska-Nowak et al., 2019 [19].

#### 4.2.2. Molecular Analyses of Nuclear Genes Associated with Optic Nerve Atrophies (Excluding *DNAJC30*)

The NGS panel for the detection of variants in three genes (*OPA1*, *OPA3*, and *TMEM126A*) associated with autosomal dominant optic atrophy (ADOA) was performed (Genomed, Warsaw, Poland) in two patients: no. 15_17 and 21_24.

Multiplex ligation-dependent probe amplification (MLPA)-based assay for the detection of *OPA1* gene deletions and duplications (Salsa MLPA Probemix, P229 OPA1, MRC-Holland, Amsterdam, The Netherlands) was carried out in three patients: no. 15_17, 18_20, and 21_24.

SNP microarray-based on APEX technology, for detecting 122 SNPs (version 1.0) in the *OPA1* gene (Asper Biotech Ltd., Tartu, Estonia), was applied in patients no. 15_17 and 18_20. Moreover, patient no. 15_17 also underwent the NGS panel for genetically determined retinal diseases encompassing 274 genes (Genomed, Warsaw, Poland). Still, the panel version at that time did not include the *DNAJC30* gene (see Supplementary Materials S1a: List of 274 genes analyzed on NGS retinal diagnostic panel).

#### 4.2.3. *DNAJC30* Gene Analyses

The fragment encompassing the coding sequence of the single-exon gene *DNAJC30* was amplified via PCR and sequenced with the Sanger sequencing method in 43 patients (all of them excluding patients no. 20_22, 23_26, and 40_45). The *DNAJC30* gene screening was performed at two genetic centers using two primer pairs designed based on the NM_032317.3 reference sequence. The PCR using the following primers F: 5′gtttCTCTTGCACCGCCTG3′ and 5′GACCGATACTCCTGCCGTTT3′ was performed in 25 patients. The primers F: 5′CTTTACGTGACTGGCCACAG3′ and R: 5′CTATCAATGGCCAAGGGTTC3′ elongated with a universal M13 sequence tag at the 5′ ends (forward 5′TGTAAAACGACGGCCAGT3′ and reverse 5′CAGGAAACAGCTATGACC3′) were applied for the molecular testing of 20 patients. The polymerase chain reaction (PCR) conditions are available upon request. The PCR products were purified with the ExoSAP-IT kit (Exonuclease I and Shrimp Alkaline Phosphatase Cleanup for PCR products, Affymetrix, Santa Clara, CA, USA) and bidirectly sequenced using dye-terminator chemistry (v3.1BigDye^®^ Terminator Thermo Fisher Scientific, Waltham, MA, USA). The sequencing products were separated on an ABI 3130xl capillary sequencer (Applied Biosystems, Thermo Fisher Scientific).

The NGS panel for the detection of variants in 26 or 30 genes associated with optic nerve atrophy pathogenesis (two versions, both including the *DNAJC30* gene) (Genomed, Warsaw, Poland) was performed in three patients: no. 20_22, 23_26, and 40_45 (see Supplementary Material S1b: List of genes analyzed on NGS panel for optic nerve atrophy (OKUA-NGS)).

The novel *DNAJC30* gene substitution, as well as two ultra-rare variants, were cross-checked with the following databases Leiden Open Variation Database (LOVD), Varsome (Varsome The Human genomics community), ClinVar, and GnomAD Browser (Genome Aggregation Database). We annotated the novel variant against the *DNAJC30* gene reference sequence NM_032317.3, following the nomenclature guidelines of the Human Genome Variation Society (HGVS, http://varnomen.hgvs.org/, accessed on 10 May 2023). The pathogenicity of the identified variants was assessed according to the American College of Medical Genetics and Genomics (ACMG) classification [20].

In silico analysis was performed to predict the possible effects of the identified novel or rare missense variants on protein function. The following tools were used: SIFT (Sorting Intolerant from Tolerant, https://sift.bii.astar.edu.sg/), PROVEAN (Protein Variation Effect Analyzer, http://provean.jcvi.org/), and PolyPhen-2 (Polymorphism Phenotyping v.2, http://genetics.bwh.harvard.edu/pph2/) (accessed on 10 July 2023).

## 5. Conclusions

To summarize, our group of patients affected with *DNAJC30* gene variants, encompassing 46 patients of Polish origin, is the largest cohort of patients with LHONAR1 described to date. Based on our data, the autosomal recessive form of LHON is more frequent than the mitochondrial form in Polish patients. We determined the substitution c.152A>G as the most common variant in the *DNAJC30* gene among Polish patients. Moreover, through the identification of a novel variant c.293A>G, p.(Tyr98Cys), this study broadens the mutation spectrum of probable pathogenic *DNAJC30* variants.

## Figures and Tables

**Table 1:** Clinical symptoms in 46 Polish patients affected with the *DNAJC30* variants.

Patient ID	Current Age (Years)/Gender	Age of Onset (Years)	First Symptoms/(BCVA OD; OS) if Known	BCVA OD; OS	Visual Field Defect	LHON or Other Ocular Symptoms in the Family
1_1	25/M	20	Deterioration of visual acuity in the RE, later in the LE, paracentral scotoma	0.04; 0.01	Irregular visual field loss	No
2_2	28/M	26	Deterioration of visual acuity in the LE, and after one month in the RE	0.1; Counting fingers	Irregular visual field loss	Blind uncle (mother’s brother)
3_3	12/M	11	Deterioration of visual acuity in both eyes, swelling of the optic nerve discs/(counting fingers)	0.1; 0.1	No data	No
4_4	24/M	21	Deterioration of visual acuity, especially in the LE	0.04; Counting fingers	Irregular visual field loss	Significantly reduced visual acuity in the deceased cousin (mother’s brother’s son)
5_5	17/F	6	Deterioration of visual acuity in both eyes	0.2; 0.25	No data	LHONAR1 confirmed in the brother no. 5_6
5_6	12/M	6	Deterioration of visual acuity in the LE, and then in the RE, color vision deterioration/(0.25)	1; 0.02	Irregular visual field loss	LHONAR1 confirmed in the sister no. 5_5
6_7	15/M	14	Deterioration of visual acuity in both eyes	0.02; 0.02	Centrocecal scotoma	No
7_8	20/M	19	Deterioration of visual acuity in the LE, and after two months in the RE	0.9; 0.01	Centrocecal scotoma	No
8_9	49/M	16	Fast and progressive narrowing of the visual field, deterioration of visual acuity/(0.04)	0.5; 0.15	Massive irregular visual field loss	LHONAR1 confirmed in the sister 8_10
8_10	47/F	22	Sudden blindness in both eyes upon awaking, loss of vision in both eyes within 2 weeks	Light perception	No data	LHONAR1 confirmed in the brother no. 8_9
9_11	48/M	47	Deterioration of visual acuity in the RE, and later in the LE/(Counting fingers; 0.3)	no data	no data	No
10_12	16/M	15	Progressive deterioration of visual acuity in the LE, and later in the RE, started after being hit in the face with a ball, color vision deterioration/(0.7; 0.3)	0.02	Centrocecal scotoma, deeper in the right eye	No
11_13	15/M	14	Deterioration of visual acuity in both eyes	0.8	No data	High myopia in the father and myopia in the sister
12_14	25/M	15	Deterioration of visual acuity in both eyes	0.1	Massive loss of visual field	Similar symptoms in the cousin, whose parents are siblings of the patient’s parents
13_15	32/M	30	Deterioration of visual acuity in the LE, and 2 weeks later in the RE	0.06; 0.4	Centrocecal scotoma, visual field disorders	No
14_16	22/M	16	Progressive deterioration of visual acuity in the LE, and 2 months later in the RE/(0.2; 0.05)	0.2 0.05	Centrocecal scotoma, visual field disorders	No
15_17	32/M	18	Progressive deterioration of visual acuity in the LE, and 2–3 months later in the RE/(0.06; 0.02)	0.1; Hand movements	Deep centrocecal scotoma	Significant visual impairment in grandmother’s 5 brothers
16_18	37/M	28	Deterioration of visual acuity in the RE (0.02), and 4 months later in the LE/(0.04)	0.5; 0.1	irregular central and paracentral visual field loss	The episode of sudden blindness for six months in the brother (at the age of 25)
17_19	25/M	17	Deterioration of visual acuity and centrocecal scotoma in RE, and 3 months later in the LE	0.1; 1	Centrocecal scotoma, visual field disorders	No
18_20	26/M	16	Sudden deterioration of visual acuity in the RE and 4 weeks later in the LE/(0.02; 0.06)	normal vision	Centrocecal scotoma	No
19_21	43/F	20	Deterioration of visual acuity in both eyes	0.41; Counting fingers	Profound visual field loss, especially central	Similar symptoms in the proband’s brother
20_22	31/M	22	Deterioration of visual acuity RE, and after 3–4 days in the LE/(0.1; 0.2)	0.3; 0.4	Central scotoma, irregular visual field loss	No
21_23	19/M	14	Progressive deterioration of visual acuity in the LE, loss of vision in both eyes within one month	1; 0.03	Irregular visual field loss: central in the left eye, peripheral in the right eye	LHONAR1 confirmed in the brother no. 21_24.
21_24	16/M	8	Deterioration of visual acuity in both eyes/(0.2; 0.6)	0.8	Deep, irregular visual field loss; Astigmatism	LHONAR1 confirmed in the brother no. 21_23
22_25	31/M	24	Deterioration of visual acuity and centrocecal scotoma in the RE, and 3 months later in the LE/(light perception)	1	Centrocecal scotoma	No
23_26	29/M	28	Progressive deterioration of visual acuity in the RE, and 5 months later in the LE	Counting fingers	Irregular visual field loss	No
24_27	40/F	29	Deterioration of visual acuity in RE and then in LE/(counting fingers; 0.1)	Counting fingers; 0.05	Centrocecal scotoma	No
25_28	34/M	22	Deterioration of visual acuity in both eyes/ (counting fingers)	0.1; 0.1	Centrocecal scotoma	No
26_29	27/M	20	Deterioration of visual acuity in the RE, and after a few months in the LE/(hand movements; 0.9)	0.1; 0.4	Centrocecal scotoma	No
27_30	35/M	28	Deterioration of visual acuity in the RE and after two weeks in the LE/(counting fingers; 0.4)	0.1; 0.07	Centrocecal scotoma	No
28_31	37/M	36	Slight deterioration of vision in both eyes/(0.8; 0.6)	0.8; 0.6	Centrocecal scotoma	LHONAR1confirmed in the brother no. 28_32
28_32	31/M	19	Deterioration of vision in both eyes/(counting fingers)	0.02; 0.03	Centrocecal scotoma	LHONAR1 confirmed in the brother no. 28_31
29_33	30/M	24	Deterioration of visual acuity in RE and then in the LE/(0.1; counting fingers)	0.15; 0.2	Centrocecal scotoma	No
30_34	33/M	18	Deterioration of visual acuity in the RE and after two years in the LE/(counting fingers)	0.16	Centrocecal scotoma, visual field disorders	No
31_35	35/M	Unknown	No data	0.31; 0.05	Centrocecal scotoma	LHONAR1 confirmed in the brother no. 31_36
31_36	37/M	29	Deterioration of visual acuity in the RE and after a few weeks in LE/(0.03; counting fingers)	0.01; Counting fingers	Centrocecal scotoma	LHONAR1 confirmed in the brother no. 31_35
32_37	26/M	23	Deterioration of visual acuity in the RE and after two years in the LE	1; 0.2	Centrocecal scotoma, visual field disorders	No
33_38	26/M	15	Deterioration of vision in both eyes/(0.31)	1	Centrocecal scotoma, visual field disorders	No
34_39	33/F	21	Deterioration of vision in both eyes/(0.8; 1)	0.62; 0.83	Centrocecal scotoma, visual field disorders	No
35_40	14/M	14	Deterioration of visual acuity in the LE and after two months in the RE/(counting fingers; 0.1)	0.8; 0.1	Centrocecal scotoma	No
36_41	13/M	13	Deterioration of visual acuity in the RE and then in the LE	0.04; 0.125	Massive irregular visual field loss	No
37_42	15/M	14	Deterioration of vision in both eyes	0.02; 0.125	Centrocecal scotoma	The same symptoms in the cousin (the father’s brother’s daughter)
38_43	21/M	16	Significant, temporal deterioration of vision after multiple head injuries (kick-boxing)/(0.06)	0.2; 0.31	Centrocecal scotoma	No
39_44	17/M	15	Blow-out orbital fracture of RE, deterioration of vision in RE, and after three months in LE	0.05; 0.1	Centrocecal scotoma	No
40_45	70/M	68	Deterioration of visual acuity in the LE and after one year in the RE, color vision deterioration/(0.05; 0.06)	0.04	No data	No
41_46	28/M	28	Progressive deterioration of visual acuity in the LE, and after a few weeks in RE/(0.1; 0.08)	0.1	Centrocecal scotoma	No

BCVA—best corrected visual acuity; OD—right eye; OS—left eye; M—male; F—female.

**Table 2:** *DNAJC30* gene variants identified in the group of 46 Polish patients.

Genotype		Number of Patients	Patient ID
Allele 1	Allele 2		
c.130_131delTC	c.152A>G	2	1_1; 22_25
c.152A>G	c.152A>G	41	2_2; 3_3; 4_4; 5_5; 5_6; 6_7; 7_8; 8_9; 8_10; 9_11; 10_12; 11_13; 12_14; 13_15; 14_16; 15_17; 16_18; 17_19; 18_20; 19_21; 20_22; 21_23; 21_24; 23_26; 24_27; 25_28; 26_29; 27_30; 29_33; 30_34; 31_35; 31_36; 32_37; 33_38; 34_39; 36_41; 37_42; 38_43; 39_44;40_45; 41_46
c.152A>G	c.293A>G ^1^	1	35_40
c.152A>G	c.293A>C	2	28_31; 28_32

^1^ A novel variant.

## Data Availability

The data used in this study is available from the corresponding author upon request.

## Supplementary Materials

The following supporting information can be downloaded at: https://www.mdpi.com/article/10.3390/ijms242417496/s1.

## References

[1] Shemesh A., Sood G., Margolin E. (2002). Leber Hereditary Optic Neuropathy (LHON).

[2] Man P.Y.W., Turnbull D.M. (2002). Leber Hereditary Optic Neuropathy. J. Med. Genet..

[3] Yu-Wai-Man P., Votruba M., Burté F., La Morgia C., Barboni P., Carelli V. (2016). A neurodegenerative perspective on mitochondrial optic neuropathies. Acta Neuropathol..

[4] Sundaramurthy S., SelvaKumar A., Ching J., Dharani V., Sarangapani S., Yu-Wai-Man P. (2021). Leber hereditary optic neuropathy-new insights and old challenges. Graefes Arch. Clin. Exp. Ophthalmol..

[5] Yu-Wai-Man P., Votruba M., Moore A.T., Chinnery P.F. (2014). Treatment strategies for inherited optic neuropathies: Past, present and future. Eye (Basingstoke).

[6] Yu-Wai-Man P., Chinnery P.F., Adam M.P., Feldman J., Mirzaa G.M., Pagon R.A., Wallace S.E., Bean L.J.H., Gripp K.W., Amemiya A. (2000). Leber Hereditary Optic Neuropathy. GeneReviews^®^.

[7] Catarino C.B., Von Livonius B., Priglinger C., Banik R., Matloob S., Tamhankar M.A., Castillo L., Friedburg C., Halfpenny C.A., Lincoln J.A. (2020). Real-World Clinical Experience with Idebenone in the Treatment of Leber Hereditary Optic Neuropathy. J. Neuro-Ophthalmol..

[8] Stenton S.L., Tesarova M., Sheremet N.L., Catarino C.B., Carelli V., Ciara E., Curry K., Engvall M., Fleming L.R., Freisinger P. (2022). DNAJC30 defect: A frequent cause of recessive Leber hereditary optic neuropathy and Leigh syndrome. Brain.

[9] Stenton S.L., Sheremet N.L., Catarino C.B., Andreeva N.A., Assouline Z., Barboni P., Barel O., Berutti R., Bychkov I., Caporali L. (2021). Impaired complex I repair causes recessive Leber’s hereditary optic neuropathy. J. Clin. Investig..

[10] Lenaers G., Beaulieu C., Charif M., Gerber S., Kaplan J., Rozet M. (2023). Autosomal recessive Leber hereditary optic neuropathy, a new neuro-ophthalmo-genetic paradigm. Brain.

[11] Magrinelli F., Cali E., Braga V.L., Yis U., Tomoum H., Shamseldin H., Raiman J., Kernstock C., Rezende Filho F.M., Barsottini O.G.P. (2022). Biallelic Loss-of-Function *NDUFA12* Variants Cause a Wide Phenotypic Spectrum from Leigh/Leigh-Like Syndrome to Isolated Optic Atrophy. Mov. Disord. Clin. Pract..

[12] Gerber S., Ding M.G., Gérard X., Zwicker K., Zanlonghi X., Rio M., Serre V., Hanein S., Munnich A., Rotig A. (2017). Compound heterozygosity for severe and hypomorphic *NDUFS2* mutations cause non-syndromic LHON-like optic neuropathy. J. Med. Genet..

[13] Gerber S., Orssaud C., Kaplan J., Johansson C., Rozet J.M. (2021). Mcat mutations cause nuclear lhon-like optic neuropathy. Genes.

[14] Zawadzka M., Krygier M., Pawłowicz M., Wilke M.V.M.B., Rutkowska K., Gueguen N., Desquiret-Dumas V., Klee E.W., Schimmenti L.A., Sławek J. (2022). Expanding the phenotype of *DNAJC30*-associated Leigh syndrome. Clin. Genet..

[15] Nesti C., Ticci C., Rubegni A., Doccini S., Scaturro G., Vetro A., Guerrini R., Santorelli F.M., Procopio E. (2023). Additive effect of *DNAJC30* and *NDUFA9* mutations causing Leigh syndrome. J. Neurol..

[16] Zaslavsky K., Margolin E.A. (2021). Leber’s Hereditary Optic Neuropathy in Older Individuals Because of Increased Alcohol Consumption During the COVID-19 Pandemic. J. Neuroophthalmol..

[17] Dimitriadis K., Leonhardt M., Yu-Wai-Man P., Kirkman M.A., Korsten A., De Coo I.F., Chinnery P.F., Klopstock T. (2014). Leber’s hereditary optic neuropathy with late disease onset: Clinical and molecular characteristics of 20 patients. Orphanet J. Rare Dis..

[18] Pfeiffer M.L., Hashemi N., Foroozan R., Lee A.G. (2013). Late-onset Leber hereditary optic neuropathy. Clin. Exp. Ophthalmol..

[19] Piotrowska-Nowak A., Kosior-Jarecka E., Schab A., Wrobel-Dudzinska D., Bartnik E., Zarnowski T., Tonska K. (2019). Investigation of whole mitochondrial genome variation in normal tension glaucoma. Exp. Eye Res..

[20] Richards S., Aziz N., Bale S., Bick D., Das S., Gastier-Foster J., Grody W.W., Hegde M., Lyon E., Spector E. (2015). Standards and guidelines for the interpretation of sequence variants: A joint consensus recommendation of the American College of Medical Genetics and Genomics and the Association for Molecular Pathology. Genet. Med..

