# Supplementary material for: DNAJC30 Gene Variants Are a Frequent Cause of a Rare Disease: Leber Hereditary Optic Neuropathy in Polish Patients
Source: Int J Mol Sci. 2023 Dec 15;24(24):17496. doi: 10.3390/ijms242417496 (PMC10743999; doi:10.3390/ijms242417496)
Supplement: Supplementary file 1 [file ijms-24-17496-s001.zip › ijms-2752625-supplementary.pdf]

Supplementary Material S1a. List of 274 genes analyzed on NGS retinal diagnostic panel

ABCA4, ABCC6, ABHD12, ACBD5, ACO2, ADAM9, ADAMTS18, ADGRA3, ADGRV1, ADIPOR1, AFG3L2, AGBL5, AHI1, AHR, AIPL1, ALMS1, ARHGEF18, ARL2BP, ARL3, ARL6, ARMS2, ARSG, ASRGL1, ATF6, ATXN7, BBIP1, BBS1, BBS10, BBS12, BBS2, BBS4, BBS5, BBS7, BBS9, BEST1, C12orf65, C1QTNF5, C2, C2orf71, C3, C8orf37, CA4, CABP4, CACNA1F, CACNA2D4, CAPN5, CC2D2A, CCT2, CDH23, CDH3, CDHR1, CEP164, CEP19, CEP250, CEP290, CEP78, CERKL, CFAP410, CFB, CFH, CHM, CIB2, CLCC1, CLN3, CLRN1, CLUAP1, CNGA1, CNGA3, CNGB1, CNGB3, CNNM4, COL11A1, COL2A1, COL9A1, CRB1, CRX, CSPP1, CTNNA1, CYP4V2, DFNB31, DHDDS, DHX38, DMD, DRAM2, DTHD1, EFEMP1, ELOVL1, ELOVL4, EMC1, ERCC6, ESPN, EXOSC2, EYS, FAM161A, FBLN5, FLVCR1, FSCN2, FZD4, GDF6, GNAT1, GNAT2, GNB3, GNPTG, GPR179, GRK1, GRM6, GUCA1A, GUCA1B, GUCY2D, HARS, HGSNAT, HK1, HMCN1, HMX1, HTRA1, IDH3B, IFT140, IFT172, IFT27, IFT81, IMPDH1, IMPG1, IMPG2, INPP5E, INVS, IQCB1, ITM2B, JAG1, KCNJ13, KCNV2, KIAA1549, KIF11, KIZ, KLHL7, LAMA1, LCA5, LRAT, LRIT3, LRP5, LTBP2, LZTFL1, MAK, MAPKAPK3, MERTK, MFN2, MFRP, MFSD8, MKKS, MKS1, MTTP, MVK, MYO7A, NBAS, NDP, NEK2, NEUROD1, NMNAT1, NPHP1, NPHP3, NPHP4, NR2E3, NR2F1, NRL, NYX, OAT, OFD1, OPA1, OPA3, OPN1SW, OTX2, PANK2, PAX2, PCDH15, PCYT1A, PDE6A, PDE6B, PDE6C, PDE6G, PDE6H, PDZD7, PEX1, PEX2, PEX7, PGK1, PHYH, PITPNM3, PLA2G5, PLK4, PNPLA6, POC1B, POCS, POMGNT1, PRCD, PRDM13, PRKCG, PROM1, PRPF3, PRPF31, PRPF4, PRPF6, PRPF8, PRPH2, PRPS1, RAB28, RAX2, RB1, RBP3, RBP4, RCBTB1, RD3, RDH11, RDH12, RDH5, REEP6, RGR, RGS9, RGS9BP, RHO, RIMS1, RLBP1, ROM1, RP1, RP1L1, RP2, RP9, RPE65, RPGR, RPGRIP1, RPGRIP1L, RS1, RTN4IP1, SAG, SAMD11, SDCCAG8, SEMA4A, SLC24A1, SLC25A46, SLC7A14, SNRNP200, SPATA7, SPP2, TEAD1, TIMM8A, TIMP3, TLR3, TLR4, TMEM126A, TMEM216, TMEM237, TOPORS, TREX1, TRIM32, TRNT1,

TRPM1, TSPAN12, TTC8, TTLL5, TTPA, TUB, TUBGCP4, TUBGCP6, TULP1, UNC119, USH1C, USH1G, USH2A, VCAN, VPS13B, WDPCP, WDR19, WFS1, ZNF408, ZNF423, ZNF513.

Supplementary Material S1b. List of genes analyzed on NGS panel for optic nerve atrophy (OKUA-NGS)

Version of 26 genes: *ACO2, AFG3L2, ANTXR1, ATP1A3, C12orf65, CISD2, DNAJC30, DNM1L, FDXR, MCAT, MFF, MFN2, OPA1, OPA3, PDXK, PRPS1, RTN4IP1, SDHA, SLC25A46, SPG7, SSBP1, TIMM8A, TMEM126A, WFS1, YME1L1, ZNHIT*

Version of 30 genes: *ACO2, AFG3L2, ANTXR1, ATP1A3, C12orf65, CISD2, DNAJC30, DNM1L, FDXR, MCAT, MFF, MFN2, MIEF1, NBAS, NDUFS4, NR2F1, OPA1, OPA3, PDXK, PRPS1, RTN4IP1, SDHA, SLC25A46, SPG7, SSBP1, TIMM8A, TMEM126A, WFS1, YME1L1, ZNHIT*
